# The safest time to fly: pandemic response in the era of Fox News

**DOI:** 10.1007/s00148-021-00847-0

**Published:** 2021-04-24

**Authors:** Maxim Ananyev, Michael Poyker, Yuan Tian

**Affiliations:** 1 University of Melbourne, Melbourne, Australia; 2 University of Nottingham, Nottingham, England

**Keywords:** Mobility, Media bias, Fox News, COVID-19, D1, D7, I31, Z13

## Abstract

We document a causal effect of the conservative Fox News Channel in the USA on physical distancing during COVID-19 pandemic. We measure county-level mobility covering all US states and District of Columbia produced by GPS pings to 15–17 million smartphones and zip-code-level mobility using Facebook location data. Using the historical position of Fox News Channel in the cable lineup as the source of exogenous variation, we show that increased exposure to Fox News led to a smaller reduction in distance traveled and a smaller increase in the probability of staying home after the national emergency declaration in the USA. Our results show that slanted media can have a harmful effect on containment efforts during a pandemic by affecting people’s behavior.

## Introduction

Media play many important roles in people’s lives by transmitting information and shaping beliefs.^1^ Such beliefs include trust in government, trust in science, and threat perceptions, which can have behavioral implications in many contexts, including public health. In such high-stakes cases as pandemics, the influence of the media on whether people comply with policies that promote safe behaviors and limit spread of a contagious disease is especially important.

In this paper, we investigate the causal impact of slanted news media on public behavior during the COVID-19 crisis. COVID-19 is a contagious disease of the respiratory system that caused a pandemic that began in early 2020. One of the measures deemed necessary to limit the spread of the disease is physical distancing (limiting travel and person-to-person interactions) because the virus spreads through droplets from infected persons (Hatchett et al. 2007; Anderson et al. 2020; Hsiang et al. 2020). Fox News Channel (hereafter, Fox), the leading cable channel in the USA, has a well-documented conservative bias in its programming Martin and Yurukoglu (2017). During the initial days of the COVID-19 pandemic, Fox’s commentators concentrated on delivering three messages: one that emphasized potential culpability of China and Chinese government in the pandemic, one that downplayed potential dangers of the virus and suggested untested medical procedures, and one that alleged that Democrats were using the pandemic to undermine President Trump before the election. These messages could potentially affect people’s evaluation of the risk and thus their willingness to self-isolate during the crisis.

Using exogenous variation in exposure to the Fox News Channel, we document a statistically significant and economically sizable effect of Fox on physical distancing. Following Martin and Yurukoglu (2017), we exploit the exogeneity of the historical position of Fox in the cable channel lineup. This variable has been shown to be unrelated to the socio-demographic and political condition prior to the introduction of Fox and strongly predictive of actual Fox viewership once the channel is introduced. Our effects can come from three channels. First, Fox viewership directly feeds people with the three aforementioned messages. Second, the build-up of the conservative ideology can make people less willing to adopt drastic changes in their behavior and living habits. Third, conservative populations may be more susceptible to Fox’s messages.

We use internet-based location data to measure social distancing behavior. It is generally hard to directly observe people’s actions. In our case, however, we measure county-level changes in distance traveled using location data of 15–17 million smartphones provided by UNACAST and zip-code-level measures of mobility using GPS pings of smartphones of Facebook users.

In our main specification, we regress the change in physical-distancing measures on Fox exposure using the standardized position of Fox in a cable lineup. Our hypothesis is that after the declaration of a national emergency on March 13, people were likely to adopt social distancing practices, but less so for regions more exposed to Fox. Although states enacted different orders in terms of shelter-in-place practices and business operations at different times, the declaration of a national emergency is a salient landmark in governments’ campaign against COVID-19 at the national level. We interact the time-invariant Fox News channel lineup position with a dummy for post- and pre-national emergency dates. Consistent with our hypothesis, we find that before the national emergency, mobility was similar in the pre-COVID period across areas with different Fox News channel positions. After the national emergency was announced, a one-standard-deviation increase in Fox exposure led to a 0.5-percentage-point larger decline in the county-level average of distance traveled relative to the pre-COVID period and a 0.1-percentage-point larger decrease in the probability of staying at home.

We conduct various robustness checks. Our results are not driven by a particular set of states and are not explained by alternative explanations, most notably that high-Fox-exposed locations are less likely to have an employment composition favourable for work-from-home or are more rural locations. Controlling for CNN and MSNBC does not affect the Fox estimates, indicating that our effects are not through crowding out of alternative media. Our result are robust to using county-level and zip code–level Facebook data for 14 states. We also provide an event-study specification that allows us to control for the time path of the effect and estimate weekly coefficients for weeks before and after the national emergency. We find that the effect of Fox News is constant in the weeks after the national emergency was pronounced and did not diminish in the 4 weeks of the post-emergency period. Finally, we replicate our results using state-specific shelter-in-place orders. While our results hold for periods after the orders were enacted, we find that people started to self-isolate even before that. Thus, overall we think that national emergency was the most salient starting point of social-distancing.

We interpret our result as the combination of the direct information channel and the indirect effect through the interaction with built-up conservatism. We control for Republican vote shares in the 2016 election, and it does not affect the magnitude and significance of the estimated Fox exposure effect.

We also provide evidence that these differences were consequential for mortality. Specifically, we find that a one-standard-deviation increase in Fox lineup position decreased the number of COVID-related deaths by 2.2% by the end of March. This result is consistent with Bursztyn et al. (2020).

Our paper contributes to several strands of literature. First is the literature on the impact of the media. Several pieces of work have documented the impact of the media on voting outcomes (DellaVigna and Kaplan 2007; Enikolopov et al. 2011), conflict (Yanagizawa-Drott 2014), and the popularity of extreme parties (Adena et al. 2015), among others. Following (Martin and Yurukoglu 2017), we add to the literature by showing how biased media can have public health consequences, a usually non-political outcome, through changing people’s behavior. We demonstrate that in addition to shaping people’s mindsets in the long run, the information conveyed by the biased media on the interpretation of scientific advice and policies can be costly to society, especially when collective action is needed in the time of public health crises.

Second, we contribute to the literature on using granular real-time individual-level data to study people’s behavior. Researchers have used cell phone location data to measure commuting and economic activities (Kreindler and Miyauchi 2019) and segregation (Athey et al. 2019); cellphones’ call data to investigate the impact of social networks on mobility (Büchel et al. 2019; Blumenstock et al. 2019), information transmission about social distancing practices (Tian et al. 2020), and job referrals (Barwick et al. 2019); and Facebook friendship data to measure social connectedness (Bailey et al. 2018) and study its impact on disease transmission in the case of COVID-19 (Kuchler et al. 2020). This type of data is especially useful in our context, since we can directly observe people’s behavior in terms of complying with social distancing policy and track real-time changes. In addition to documenting changes in mobility before and after the declaration of a national emergency and the geographic distribution of mobility, we investigate the potential determinants of such geographic variation and highlight the importance of the media.

Finally, our paper adds to the rapidly growing literature on the COVID-19 pandemic, especially on determinants of physical distancing and transmission. There are also two contemporaneous papers that study the effect of Fox News exposure on public-health behaviors: Ash et al. (2020) and Simonov et al. (2020). We discuss this literature in the next section.

This paper proceeds as follows. Section 2 summarizes the literature related to COVID-19 and social distancing. Section 3 introduces background information about the development of COVID-19 in the USA, policies on social distancing, and its coverage by Fox News. Section 4 describes our data. Section 5 introduces our empirical specification, identifying assumptions, and results. Section 6 concludes.

## Review of COVID-19-related literature

Our paper contributes to a rapidly developing social-science literature on COVID-19. The first set of literature discusses the effectiveness of government policies in shaping the responses to the pandemic. Qiu et al. (2020) analyse the containment policies in China and concludes that lockdowns and quarantine were essential for slowing down the spread of the virus. Bonacini et al. (2021) propose a methodology based on structural breaks to measure the effectiveness of policy responses to COVID-19. Political cleavages have been shown to be consequential for the policy response to the pandemic. In particular, Adolph et al. (2020) demonstrate that Republican governors in the USA were slower to implement stay-at-home orders and other restrictions, and Besley and Dray (2020) show that the association between COVID-19 deaths and lockdowns is stronger in countries with free media. In our paper, we show that the policy change at the federal government level, specifically, the declaration of national emergency in the USA, combined with media consumption, had a large impact on people’s mobility reduction.

Another set of studies explore other socio-economic determinants of public-health behavior. Tian et al. (2020) and Milani (2021) show that social networks increased spillovers in social distancing behaviors. Socio-economic and demographic factors associated with self-protective behaviors have been explored by Papageorge et al. (2021) who show that low income and less flexible work arrangements are negatively associated with safe behaviors, while Chiou and Tucker (2020) show positive association between high income/high-speed internet and propensity to stay at home. These findings are consistent with Wright et al. (2020) who show that exposure to trade shocks and conservatism is negatively associated with compliance with stay-at-home orders in the USA. Egorov et al. (2020) demonstrate that counties with higher ethnic fractionalization experienced larger reductions in mobility. Messages from political leaders were also demonstrated to be consequential for safe behaviors: Ajzenman et al. (2020) and Mariani et al. (2020) show that the safe behaviors in Brazil declined in places with stronger support for President Bolsonaro once he publicly dismissed the dangers of the virus.

When it comes to partisan differences in reactions to the pandemic, Barrios and Hochberg (2020) and Gadarian et al. (2020) demonstrate a partisan gap in perceptions of risk posed by COVID-19, while Allcott et al. (2020), Andersen (2020), and Painter and Qiu (2020) show a gap in a physical distancing between places with more Republicans and places with more Democrats and suggests that partisan messaging was one of the mechanisms. While those contributions are suggestive of a causal role of conservative media, they are also consistent with a proposition, developed in a recent theoretical contribution by Gitmez et al. (2020), that people who are ex-ante less likely to comply would choose a media source that is more likely to downplay the risks of the crisis. We share the features of many of these papers by using cellphone location data to measure mobility; however, by using a plausibly exogenous variation in exposure to Fox, we causally identify the effect of media on social distancing practices. We emphasize that not only the pre-existing political views but also the flow of information through (politicized) media can shape people’s view.

A paper close to ours is Bursztyn et al. (2020) which identifies the effect of watching the most popular Fox show, Hannity, on mortality. We instead focus on behavior responses. By using much finer geographically variation (county-level and zip-code-level instead of relatively large Designated Market Area level), the timing of the declaration of national emergency, and direct measures of behavior responses, we show how exactly Fox viewership can affect efforts in combat with the infectious diseases.

Contemporaneous papers by Simonov et al. (2020) and Ash et al. (2020) use the same idea of identifying variation that we exploit (the Fox News channel position) and the SafeGraph data for social-distancing measures (we use UNACAST and Facebook data instead). There are several notable differences between our approach and that of Simonov et al. (2020) and Ash et al. (2020). First, we use the earliest available data on the Fox News Channel position (from 2005), while Simonov et al. (2020) use the data from 2015, and Ash et al. (2020) use channel positions from 2016. This difference between 2005 and 2015/2016 is important because cable networks are well aware of the influence that channel position has on the viewership and lobby providers to put their channels “lower on the dial” Snider and Hall (1998). This is especially true for Fox: as early as 2007, the movement of the channel from position 46 to position 44 in one of the Time Warner Cable markets was seen as a major win that merited inclusion in a self-congratulatory announcement by Fox.^2^ Thus, Fox News Channel position in the later years is more likely to be endogenous to the lobbying efforts of Fox leadership.^3^ Second, we make an attempt to disentangle different mechanisms. The effect of Fox exposure on social-distancing can be potentially explained by the three channels, conservatism, COVID-19-related information transmission, and the interaction of the two. The conservatism can influence social-distancing directly by making people skeptical of governmental interventions and academic experts. Because we use the historical position of Fox and control for the built-up conservatism (by including controls for the 2016 election results), we can isolate the effect of COVID-19 messaging through Fox and shedding light on the mechanisms.^4^

Another important difference between our paper and the ones by Simonov et al. (2020) and Ash et al. (2020) is that, for their measures of physical mobility, they rely on a single dataset, provided by SafeGraph company, while we rely on two independent datasets: by UNACAST and by Facebook. To be more specific, Simonov et al. (2020) use SafeGraph panel to construct Census Block-level aggregates of movement, while (Ash et al. 2020) aggregate Census Block Groups (CBG) to zip-code level and to county level. It is documented that the SafeGraph panel tends to oversample Census Block Groups with fewer people (SafeGraph itself calling the representativeness on this level “complicated”).^5^ Thus, the aggregation procedures used in those papers might lead to biases.^6^

Data that we use also have biases. Specifically, Facebook users are younger and more urban than US population.^7^ The UNACAST data oversamples the high-income neighborhoods and certain states.^8^ Given that all mobility data sources exhibit biases, it is critical not to rely on one data provider but analyze several data sources. We show that our two sources generate highly correlated measures of mobility reduction, and our empirical results are robust to using different measures.^9^

## Background: COVID-19 and Fox News Channel

### The US COVID situation and government responses

#### COVID-19 and Social-Distancing

COVID-19 is a disease of the respiratory system caused by a new coronavirus (SARS-CoV-2). The first case was reported on December 31 in Wuhan, China, and the first death from the new virus was reported in China on January 7. The virus then rapidly spread to other countries (the first case outside China was reported on January 13, 2020). The WHO declared a pandemic on March 11, 2020.^10^ The first confirmed case in the USA happened on January 21, 2020. As of April 28, 2020, the Center for Disease Control and Prevention (hereafter, CDC) reported 981,246 total cases in the USA and 55,258 deaths related to the illness.^11^ Due to its means of transmission, the CDC advised that “limiting face-to-face contact with others” was “the best way to reduce the spread of coronavirus disease.”^12^

#### National Emergency, State, and Local Stay-at-Home Orders

President Trump declared National Emergency on March 13, 2020.^13^ It was the first important signal to the whole nation that the threat is real: “’Trump said he had ordered all states to set up emergency operation centers, and urged hospitals to engage emergency operation plans.”^14^ From a policy standpoint, the most important implication of the national emergency was a fiscal one: it became easier for Federal Emergency Management Agency to transfer money to the states. It has freed up as much as $50 billion in financial resources. The importance of this move can also be perceived from how the stock market has reacted. Stock markets had their largest single-day gain since October 2008.^15^

**Fig. 1:**
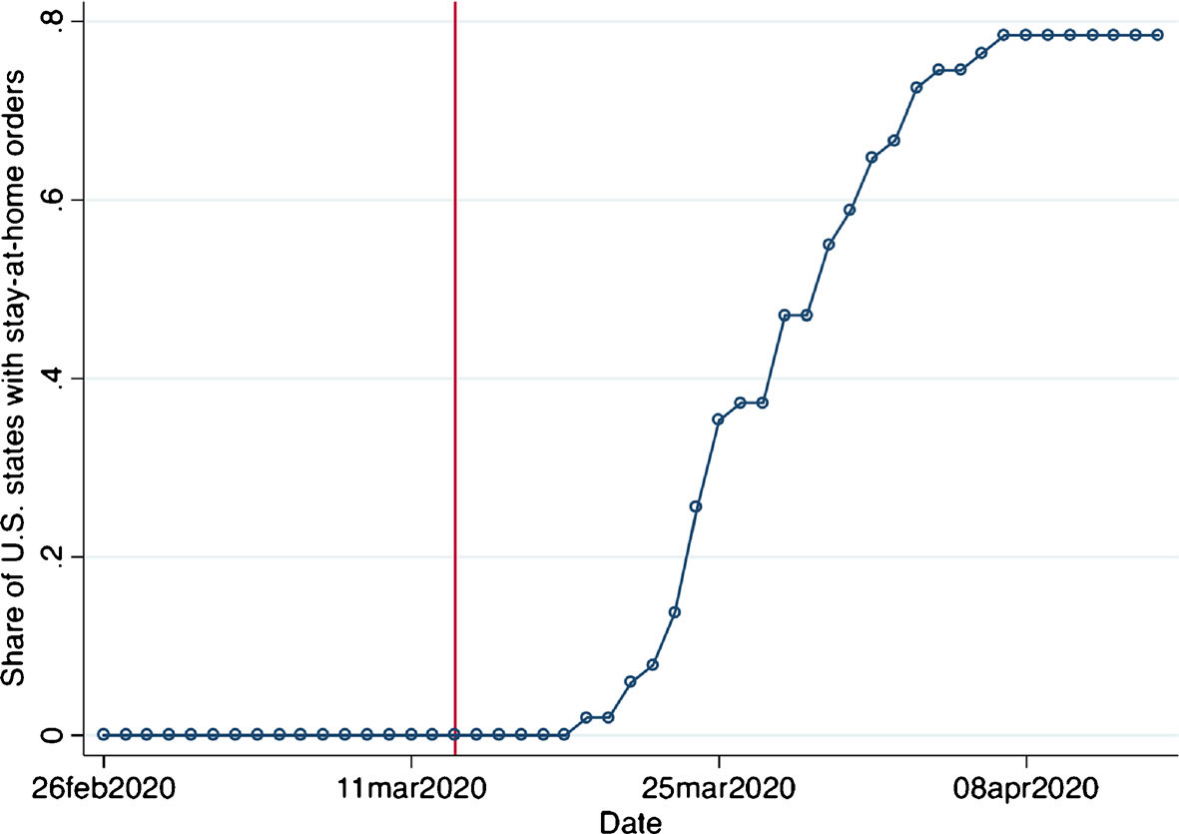
National Emergency and Stay-at-Home Orders. Note: This figure shows the share of US states that have stay-at-home orders on a particular date. All 50 states and the District of Columbia are included. Stay-at-home or shelter-in-place orders only include directives and orders, but not guidance, and the order must apply to the entire states. According to this definition, 11 states never enacted an order: Arkansas, Connecticut, Iowa, Kentucky, Nebraska, North Dakota, Oklahoma, South Dakota, Texas, Utah, and Wyoming. The vertical line represents March 13, 2020, when the USA declared a national emergency. See details of the definition at Raifman et al. (2020)

The declaration itself did not imply state-at-home mandates or any other regulations as those decisions were left to the states. Among the states, the first stay-at-home order was issued by California on March 19. This move was later followed by 39 other states (see Fig. 1). The last order was issued by South Carolina on April 7th. Eleven states (Arkansas, Connecticut, Iowa, Kentucky, Nebraska, North Dakota, Oklahoma, South Dakota, Texas, Utah, and Wyoming) did not issue shelter-in-place orders during the period that we study. Those orders differed widely in content and in how strictly they were enforced.

We use March 13 as the starting date where we expect mobility responses because it should have signaled the seriousness of the pandemic to the US population. As we demonstrate later in Fig. 3, on average the movement started to decline around that date.^16^

### Messages of the Fox News Channel

Fox News is the leading cable channel in the USA with an estimated 3.5 million prime-time viewers.^17^ During the initial days of the COVID-19 spread, Fox engaged in three major discussions on the topic: China’s culpability, COVID-19’s insubstantiality, and Democrats’ partisan interests.

First, when President Trump used the term “Chinese coronavirus,” some of his critics suggested that this term would fuel prejudice against Chinese nationals in the USA and Chinese-Americans. Some of the Fox hosts spent a significant amount of time rebutting this claim. For example, Sean Hannity said on March 12, 2020:

Over there at fake news CNN, you have fake news Jimmy Acosta, well, he’s most worried about the president’s terminology, thinking the president’s speech was racist because he said the fire started in China.^18^

Another issue was on the credibility of Chinese data. On February, 18, Laura Ingraham, the host of “Ingraham Angle” (the third most-watched Fox show), said:

All right and speaking of China, as the coronavirus spreads, the flow of reliable information from China is basically trickling to a stop, if it ever existed at all. Now why is that? And what exactly are they hiding from us?^19^

Fox personalities also discussed a specific hypothesis about the origin of the virus, suggesting that it might come from Wuhan Institute of Virology. In particular, Tucker Carlson, the host of “Tucker Carlson Tonight” (the second most-watched Fox show), said on March 12. 2020:

In fact, the outbreak may have begun not in a public meat market, but in a poorly run Chinese laboratory. Now, that’s not our theory. Anyone who raises that theory on American television is attacked as a conspiracy monger.^20^

Second, many of the Fox hosts either were dismissive towards the potential dangers of the virus or ignored it completely. On March 13 (two days after the WHO had declared a pandemic), “Fox & Friends” host Ainsley Earhardt told the viewers that it was “the safest time to fly” because “the terminals are dead.” Another Fox personality, Jeanine Pirro, the host of “Justice with Judge Jeanine,” on March 7 said: ”All the talk about coronavirus being much more deadly [than seasonal flu] does not reflect reality.”^21^ In addition, some shows spread misinformation of diagnostics and preventive methods. For example, Correspondent Geraldo Rivera suggested a simple (but lacking any scientific merit) diagnostic procedure:

If you can’t hold your breath for 10 seconds. Everyone should do that. Hold your breath for 10 seconds. If you can hold your breath for 10 seconds then you don’t have this disease.^22^

Third, a large amount of air time was devoted to accusation of the Democratic party’s “politicizing” the virus and using it opportunistically to harm the reputation of President Trump. On March 9, Fox host Sean Hannity, suggested that opponents of the president were “scaring the living hell out of people.”^23^ Laura Ingraham, the host of “Ingraham Angle,” said on February 25:

After their politically disastrous impeachment and the fierce intraparty fighting ... Democrats needed to change the subject and fast. So, like the Coronavirus itself, Democrats and friends moved to quickly infect the political discussion with viral recriminations.^24^

After the declaration of national emergency by President Trump on March 13, 2020, the messaging of Fox shifted towards more emphasis on the importance of distancing and other preventive measures, but not entirely.^25^ In addition, the initial period of partisan messaging could have influenced the attitudes of Fox viewers in a way that later shifts could not completely revert due to the confirmation bias (Nickerson 1998).

The coronavirus coverage by Fox was different from that by other major cable channels. The most popular host of MSNBC (the second most-watched cable channel in the USA), Rachel Maddow covered the spread of the virus, both internationally and in the USA, and criticized the Republican administration for lack of testing capacity and other issues.^26^ CNN (the third most-watched cable channel) largely focused on reporting facts, with occasional criticism of some of Trump’s epidemiological claims.^27^

**Fig. 2:**
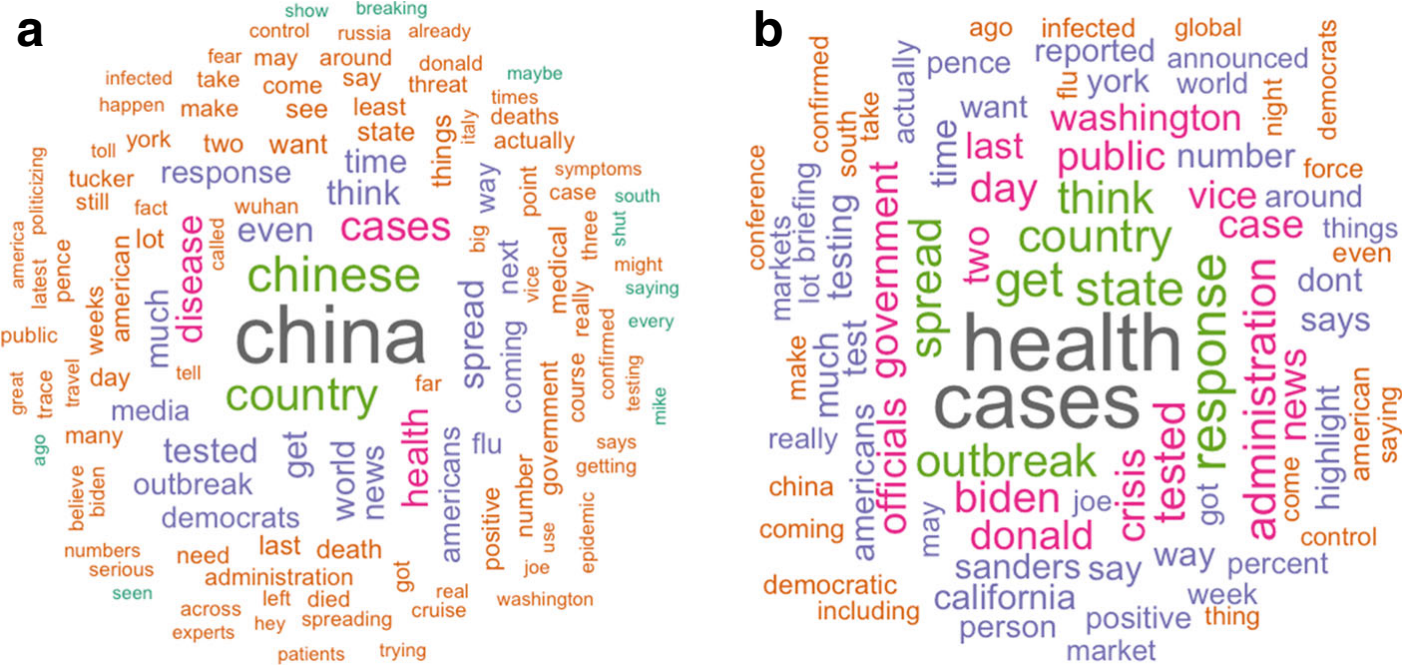
Word Clouds of COVID-19 Coverage for Fox News and MSNBC. Note: This figure shows word clouds of COVID-19 coverage from the three most-watched Fox shows (Hannity, Tucker Carlson Tonight, and Ingraham Angle) on the left (panel A) and MSNBC on the right (panel B). Transcripts are from February 1, 2020, to March 12, 2020, downloaded via LexisNexis. To build the word clouds, we selected only paragraphs containing the word “coronavirus” and removed common English stop-words as well three common words on both Fox and MSNBC (“president,” “Trump,” and “people”). We also removed words that are not informative about the tone of the coverage, like “united,” “states,” “white,” and “house.” We built word clouds with remaining words. **a** Fox News Channel **b** MSNBC

To illustrate some of the distinct features of Fox coronavirus coverage, panel A of Fig. 2 plots the word-cloud of paragraphs including the word “coronavirus” constructed from LexisNexis transcripts of top-3 Fox shows (“Hannity,” “Tucker Carlson Tonight,” and “Ingraham Angle”) as well as a similar word cloud of MSNBC transcripts from February 1 to March 12 in panel B. For both networks, we excluded the three most common terms: “President,” “Trump,” and “people.” We see that the most common words in MSNBC coverage were “health” and “cases,” while for the top Fox commentators, two most common words were “China” and “Chinese.”

It is important to note that, while other contemporaneous studies on this topic emphasized misinformation as the main channel of the effect, our reading of Fox News coverage suggests that pivot to the culpability of China (which by itself might not necessarily be a misinformation) was also important. These discussions do not necessarily constitute misinformation, but they can be powerful in shaping people’s behavior as well through the mechanism of priming (Domke et al. 1998; Druckman 2004). Because of cognitive limitations, messages from the media influence people’s “top-of-the-head considerations” Zaller et al. (1992), thus emphasizing “China” in the context of COVID-19 makes other mental constructs (for example, those of safety measures) less salient. This is why talking about China instead of talking about safety can decrease people’s compliance with the safety measures.

### The effect of Fox News Channel on the compliance with social-distancing

**Fig. 3:**
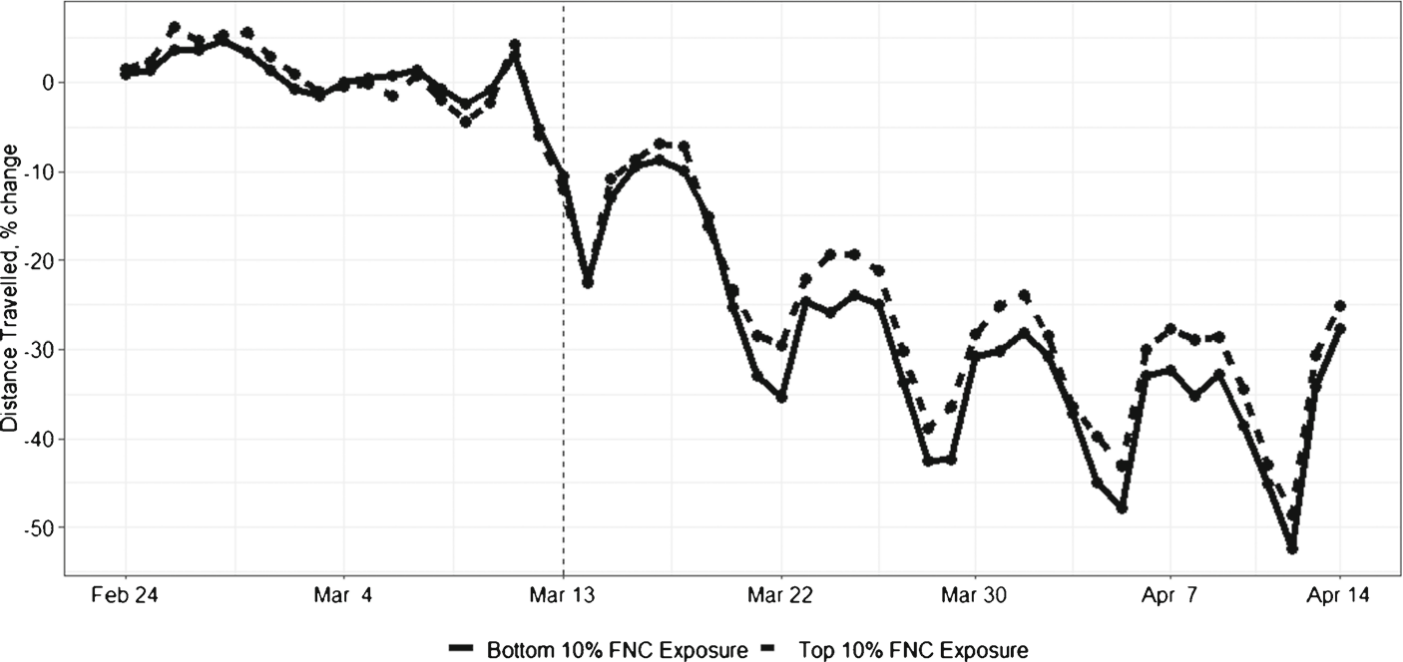
Fox News and Social Distancing. Note: This figure shows the changes in daily distance traveled. The solid line shows changes in counties in the bottom 10% of Fox News exposure (i.e., higher Fox News Channel number, channel positions 64 to 95). The dashed line shows changes in counties in the top 10% of the Fox News exposure (i.e., lower Fox News Channel number, channel positions 1 to 24). The vertical dashed line represents the announcement of a national state of emergency on March 13th

As a preview of our main result, we present visual evidence on how exposure to Fox affected compliance with social-distancing. Figure 3 plots changes in daily distance traveled for the top 10 percentile of US counties in terms of channel position of Fox (dashed line) and that of the bottom 10 percentile (solid line).^28^ There are three observations from the graph. First, before the National Emergency (vertical dashed line), both types of counties did not change the patterns of mobility compared to the pre-COVID period. Second, after March 13, both groups reduced mobility. Third, afterward, counties with lower channel positions (solid line) experienced a larger decline in the daily distance traveled than those with the higher channel position, highlighting the role of Fox exposure.

## Data and measurement: Fox News exposure and social distancing

### Exogenous variation in fox news exposure

We first construct the measure of exposure to Fox. Fox viewership is correlated with political preferences, which can potentially bias our estimate of the effect of Fox viewership on social distancing. For example, since many of the Fox hosts are conservative, its viewers might be inherently more likely to view government measures with suspicion, which might reduce their compliance. Also, given the well-documented urban/rural ideological divide in the USA, it is likely the people from rural counties watch Fox more and are more limited in how much travel they can avoid.

Instead of using actual viewership, we use an exogenous variation in exposure to Fox: the position of Fox News in the cable lineup. Fox was launched in 1996 and quickly expanded its geographic coverage through bilateral negotiations with local cable providers. As a result of those negotiations, those providers started offering Fox as a part of their packages, usually replacing one of their channels with the goal to minimize the change in the existing lineup and not to disrupt the experience of the viewers. This process created quasi-experimental variation in Fox exposure. When Fox has a larger number in the cable lineup position, people are less likely to watch it because it takes more effort to move to this channel. See detailed discussions in Martin and Yurukoglu (2017).^29^

We obtain zip-code-level average historical (2005) position of Fox by Nielsen from Ash and Poyker (2019). Fox became increasingly conservative afterwards and started to lobby lower channel positions as early as in 2007. County-level measures of exposure are aggregates of zip-code-level ones using population weights. Fox Newa channel positions vary from #1 in cable lineup to #95, and its standard deviation is about 15 channels.^30^Martin and Yurukoglu (2017) demonstrate that at the zip-code level, Fox News Channel position is not predicted by the 1996 Republican voting share or electoral contributions and is not explained by predicted voting outcomes and viewership using the 2010 demographics. We find similar results using the county-level voting shares, as shown in Table 1. In addition, Table 1 includes an array of balance tests, where we regress 2010 demographic and socio-economic variables on Fox News Channel position in cable lineup in 2005. All estimates are not statistically different from zero. We also show that Fox News channel positions in 2005 are not correlated with pre-COVID mobility and pre-National-Emergency COVID cases the deaths. On the other hand, Fox exposure is correlated with the Republican vote share in 2012 and 2016, suggesting that the channel positions in 2005 affected conservatism in later years.^31^

**Table 1:** Balance tests

	(1)	(2)	(3)
	Coefficient	S.E.	*p*-value
Socio-demographic controls:			
Population	0.244	(0.146)	[0.101]
Poverty	–0.013	(0.009)	[0.189]
Urban/rural	0.030	(0.018)	[0.114]
Share nonwhite	0.155	(0.385)	[0.690]
Dom. migration	0.356	(0.320)	[0.271]
No high school	0.046	(0.139)	[0.739]
Median income	0.011	(0.007)	[0.101]
Workable-at-home jobs, share	0.007	(0.008)	[0.394]
Workable-at-home jobs, share (wage weights)	0.008	(0.010)	[0.413]
Workable-at-home jobs alt., share	0.006	(0.007)	[0.414]
Workable-at-home jobs alt., share (wage weights)	0.007	(0.009)	[0.454]
Means of transport. to work (car, truck, or van) 2005–2009, share	0.00	(0.002)	[0.843]
Means of transport. to work (public w/o taxi) 2005–2009, share	0.002	(0.001)	[0.154]
Republican vote shares:			
Presidential election Republican vote share, 2016	–0.684**	(0.318)	[0.037]
Presidential election Republican vote share, 2012	–0.787*	(0.418)	[0.066]
Presidential election Republican vote share, 1996	–0.108	(0.192)	[0.576]
Pres. el. Rep. vote share (predicted from demog), 1996	0.014	(0.110)	[0.903]
Pre-COVID mobility:			
Differences in daily distance traveled	0.001	(0.065)	[0.982]
Baseline probability staying at home	0.001	(0.002)	[0.659]
Baseline distance traveled	0.018	(0.030)	[0.550]
Pre-National Emergency COVID cases and deaths			
Pre-March 13 number of COVID cases per capita	0.027	(0.039)	[0.491]
Pre-March 13 number of COVID deaths per capita	–0.005	(0.004)	[0.232]

Column 1 contains coefficients from the bivariate regression of Fox News Channel position on various outcomes. All regressions include state fixed effects. Column 2 reports standard errors clustered on the state. Column 3 reports *p*-values. None of the regressions are significant at any conventional level. Data on workable-at-home jobs is from Dingel and Neiman (2020). County-level voting data is from https://github.com/tonmcg/County_Level_Election_Results_12-16. Socio-economic data (except for “nonwhite” variable) is from https://www.ers.usda.gov/data-products/county-level-data-sets/county-level-data-sets-download-data/. The share of nonwhite population is from http://library.duke.edu/data/collections/popest. Means of transportation to work data is from US Census Bureau (https://www.census.gov/library/publications/2011/compendia/usa-counties-2011/basic-info-file-formats.html). Numbers of COVID cases and deaths are from https://coronavirus.jhu.edu/

### Smartphones data on people’s mobility

The county-level estimates of reductions in mobility come from the New York–based technology company UNACAST, inc (Unacast 2020). Using the GPS locations, an identifier (smartphone) is assigned to a county with the largest total duration of stay. There are 15–17 million identifiers for each day in the dataset, from February 24, 2020, to April 14, 2020, and the total distance traveled per device is then averaged at the county level.^32^ To take into account the baseline differences in mobility across regions, each weekday is assigned a baseline distance traveled, using the same weekday during the four weeks before March 8, 2020 (a date that is coded as the start of COVID-19 outbreak in the USA). Then, the reduction in distance traveled in a day is measured as the percent reduction between the current date and the baseline weekday. The raw variation in average changes in daily distance traveled (post-March 13, 2020) can be found in Ananyev et al. (2021) Figure A.2.

The zip-code-level mobility measure is constructed using data from Facebook’s Data for Good.^33^ In contrast to the UNACAST dataset, the Facebook data starts on March 10, 2020 and covers only 327 counties on the east coast and west coast of the USA, located in District of Columbia and 14 states.^34^ There are about 4.16 million devices per day.^35^ With information from people using Facebook on their mobile phones with Location History enabled, a person’s movement between two time windows is measured as tile-to-tile movements, where a time-window is an 8-hour period and a tile is a 10 km by 10 km ground square.^36^ After assigning tiles to zip codes/counties, we construct two measures using these movement vectors: (i) the probability of staying in the same tile, which we call “staying at home,” and (ii) total distance traveled.^37^ Since there are three time windows per day, we take the mean of the three observations. Pre-COVID period is defined as the 45 days prior to March 10, 2020, and both measures are constructed for this baseline period. Although the baseline data is constant at each tile-to-tile vector, mobility measures at different dates can still have different baseline values since a vector is only recorded if more than 10 users made the move. In addition to the mobility information, we also construct the total Facebook population at the zip code and at the county level.

### Other factors affecting mobility

There are several factors other than media viewership that could potentially affect the extent of social distancing practices. Importantly, some jobs can be more easily switched to the online mode than others. Thus, depending on the industry where people work, a region’s compliance with social distancing policy can vary. We computed county-level shares of employment in workable-at-home industries using data from Dingel and Neiman (2020). Other county-level measures include voting outcomes in the Presidential elections of 2012 and 2016 and socio-economic and demographics variables in 2010. The details of the data sources and summary of statistics can be found in Table 1.

## Empirical specification and results

### Empirical specification

The objective of the empirical exercise is to identify the effect of exposure to Fox on social distancing after the National Emergency was announced on March 13th. The pre-March 13th observations are used to test pre-trends. Our main specification is the following county-date panel regression:

1 $$  \mathit{SD}_{i(s)t} = \beta_{1}\mathit{FNCP}_{i(s)} \times \text{Before}_{t} + \beta_{2}\mathit{FNCP}_{i(s)} \times \text{After}_{t} + X_{i(s)}{\Gamma} + \mu_{s} + \lambda_{t} + \epsilon_{i(s)t}, $$

where *SD*_*i*(*s*)*t*_ is a measure of social-distancing in a county *i* located in state *s* on date *t*, *FNCP*_*i*(*s*)_ is the 2005 Fox position in channel lineup, and Before_*t*_ (After_*t*_) is a dummy equal to one for dates before (after) the national emergency. *FNCP*_*i*(*s*)_ is normalized to have a mean of zero and a standard deviation of one, and a larger *FNCP*_*i*(*s*)_ is associated with a smaller exposure to Fox News. We control for state (*μ*_*s*_) and date (*λ*_*t*_) fixed effects. Vector *X*_*i*(*s*)_ includes a set of county-level demographic and economic controls such as population density and poverty rate. Standard errors are clustered at the state level.^38^

The coefficient of interest *β*_2_ captures the effect of Fox on social distancing after the National Emergency was announced. We expect it to be negative: counties with larger Fox lineup positions have a larger decrease in the daily distance traveled relative to their pre-COVID baseline. *β*_1_ represents the effect of Fox on social distancing before the National Emergency was announced, and we expect it to be zero, indicating that counties with difference Fox exposure did not exhibit differential social distancing behaviors in the pre-period.^39^ Since our Fox News Channel lineup is measured in 2005, which is earlier than our study period (2020), we are eventually studying the heterogeneous effect of built-up Fox exposure on people’s behavioral choices, given that there is a national policy advocate.^40^

In addition to the demographic and economic controls mentioned earlier, we take into account the potential confounding effect of the industrial composition on the relationship between Fox exposure and social distancing behaviors. Regions with more Fox exposure may have a particular employment mix, and as shown in Dingel and Neiman (2020), industries and occupations differ in their workability-at-home. We control for it directly in our regressions.

Fox exposure can affect the degree of conservatism, which can directly affect the social distancing behavior if a conservative population has different preferences or different constraints, and indirectly, through the interpretation of the COVID-related messages conveyed by Fox. These effects are on top of direct information feeds by Fox that may affect all of its audience, irrespective of their ideology. To separate (1) conservatism, (2) information, and (3) the interaction of the two, we also experiment with directly controlling for the county-level Republican vote shares in the 2012 and 2016 presidential elections and the 2016 turnout rate, which act as proxies for built-up conservatism. In this case, the remaining effect of Fox on social distancing should be either through the information feed or through the interaction of information and conservatism.^41^,^42^

One might be concerned that similar to the Fox exposure, other county-level characteristics also affect the social distancing behaviors differentially *before* and *after* the declaration of national emergency. If the Fox exposure is correlated with these characteristics, omitting this differential impact may bias our estimate of the Fox coefficient. To address this concern, we also consider a specification where we add the interaction of the controls *X*_*i*(*s*)_ with the After dummy.

Although we include a wide variety of county characteristics, there might be unobserved variables that are correlated with Fox exposure and affect the social distancing outcome. Thus, we also present a specification where instead of the state fixed effects, we use county fixed effects. Here, both the Fox interaction with the Before dummy and the levels of county characteristics *X*_*i*(*s*)_ will be absorbed by the fixed effects.

### Main results

Table 2 shows the effect of Fox exposure on social distancing using various specifications. In panel A column 1, we estimate Eq. 1 with only state and date fixed effects. The estimand $\widehat {\beta }_{1}$ is statistically insignificant, indicating that counties did not have differential patterns in social distancing before the National Emergency. The point estimate of interest $\widehat {\beta }_{2}$ is negative and significant. It indicates that a one-standard-deviation increase in Fox News Channel lineup led to a 0.6-percentage-point larger decline in average distance traveled in a county.

**Table 2:** Effects of Fox News Channel position on reductions in mobility

	(1)	(2)	(3)	(4)	(5)	(6)	(7)
	Dependent variable: difference in daily distance traveled						
Panel A: baseline							
Fox News Channel position	0.002	0.003	0.003	0.003	0.003	0.003	0.003
x Before National Emergency	(0.0019)	(0.0018)	(0.0019)	(0.0021)	(0.0021)	(0.0021)	(0.0021)
Fox News Channel position	–0.006**	–0.005**	–0.005**	–0.005**	–0.005**	–0.005**	–0.005**
x After National Emergency	(0.0027)	(0.0025)	(0.0024)	(0.0023)	(0.0023)	(0.0024)	(0.0023)
R-squared	0.669	0.678	0.681	0.688	0.688	0.689	0.692
Observations	119,876	119,876	119,876	119,876	119,876	119,876	119,876
Panel B: with other channels							
Fox News Channel position	0.002	0.002	0.002	0.002	0.002	0.002	0.002
x Before National Emergency	(0.0020)	(0.0019)	(0.0019)	(0.0022)	(0.0022)	(0.0022)	(0.0021)
Fox News Channel position	–0.005*	–0.005*	–0.005**	–0.005*	–0.005*	–0.005**	–0.005**
x After National Emergency	(0.0028)	(0.0026)	(0.0024)	(0.0023)	(0.0023)	(0.0024)	(0.0023)
CNN channel position	–0.003	–0.003	–0.002	–0.002	–0.002	–0.002	–0.001
	(0.0019)	(0.0017)	(0.0016)	(0.0017)	(0.0017)	(0.0017)	(0.0017)
MSNBC channel position	–0.001	–0.000	0.000	0.001	0.001	0.001	0.001
	(0.0018)	(0.0016)	(0.0015)	(0.0014)	(0.0014)	(0.0014)	(0.0013)
R-squared	0.687	0.696	0.699	0.706	0.706	0.707	0.710
Observations	104,400	104,400	104,400	104,400	104,400	104,400	104,400
Panel C: with post-							
national emergency controls							
Fox News Channel position	0.002	0.002	0.002	0.002	0.002	0.002	0.002
x Before National Emergency	(0.0019)	(0.0016)	(0.0016)	(0.0015)	(0.0015)	(0.0015)	(0.0015)
Fox News Channel position	–0.006**	–0.005**	–0.005**	–0.004*	–0.004*	–0.004*	–0.004*
x After National Emergency	(0.0027)	(0.0024)	(0.0023)	(0.0023)	(0.0023)	(0.0023)	(0.0023)
R-squared	0.669	0.683	0.685	0.696	0.697	0.698	0.706
Observations	119,876	119,876	119,876	119,876	119,876	119,876	119,876
Panel D: with county FEs &							
post-national emergency controls							
Fox News Channel position	–0.008**	–0.008**	–0.008**	–0.007**	–0.006**	–0.006**	–0.007**
x After National Emergency	(0.0033)	(0.0030)	(0.0030)	(0.0027)	(0.0026)	(0.0026)	(0.0027)
FEs: County	✓	✓	✓	✓	✓	✓	✓
R-squared	0.756	0.763	0.763	0.770	0.770	0.771	0.774
Observations	119,876	119,876	119,876	119,876	119,876	119,876	119,876
Economic controls		✓	✓	✓	✓	✓	✓
Urban			✓	✓	✓	✓	✓
Population controls				✓	✓	✓	✓
Share nonwhite & migrant					✓	✓	✓
Workable-from-home emp.						✓	✓
Repub. vote share controls							✓

The explanatory variable in all panels is normalized to mean zero and standard deviation one. The dependent variable is the difference in daily distance traveled. All regressions include state and date fixed effects. Economic controls include unemployment rate, economic-dependence county indicator, poverty rate, and median income. Urban controls include eight dummies for urban-rural continuum. Population controls include population, share of population with high-school education, and county’s land area. Share nonwhite and migration controls include share of nonwhite population and net domestic migration rate. Workable-from-home employment control includes employment share in workable-from-home industries (according to Dingel and Neiman (2020)). Republican vote share controls include Republican vote share in 2012 and 2016 presidential elections, and 2016 turnout rate. In parentheses we report standard errors clustered on the state. ***p< 0.01, **p< 0.05, *p< 0.1

To put the magnitude of our results in context, the biggest decrease in distance traveled per person after March 13 happened in the District of Columbia (59%), and the smallest one—in Nevada (13%). According to the estimates of Martin and Yurukoglu (2017), moving Fox from channel 10 to channel 40 (approximately, two standard deviations) is associated with a 5-min reduction per week per person in time spent watching Fox. According to our results, when Fox is moved 30 positions higher in the cable lineup, it decreases social-distancing by one percentage point. Assuming linear marginal effects and comparing the effect size with the state-level reduction in mobility, we can roughly estimate that this effect can explain 2% and 8% of the total reduction in population movement in DC and Nevada, respectively.

In columns 2–5, we sequentially add controls for demographic and socio-economic variables. As documented in various other studies, higher education levels and higher incomes at the individual and region levels are positively associated with practicing of social distancing (Brzezinski et al. 2020, Fan et al. 2020, Mongey et al. 2020, Wright et al. 2020, among others). We control for the unemployment rate, urban dummies, economic-dependence county indicator, poverty rate, median income, population, the share of the population with a high-school education, county’s land area, the share of the nonwhite population, and net domestic migration rate. Column 6 further adds the employment share in workable-at-home jobs.^43^ The coefficient estimate for the Fox effect remains almost identical compared to column 1.

As Fox can affect the general level of conservatism of the local population, it can potentially affect people’s response towards recommendations for social distancing. Column 7 adds controls for the turnout in 2016 and Republican vote share in the 2012 and 2016 elections. We find that controlling for these conservatism proxies does not affect the coefficient estimate of Fox exposure.^44^ It suggests that our results are not driven by the accumulated Fox effect but by its immediate reaction to COVID-19, and possible by the interaction of the two.

We also want to test if the Fox effect comes from crowding out viewership of other media. If people watch less Fox and at the same time watch more of other channels such as CNN and MSNBC, our coefficient estimates may reflect the positive effect of other media instead of the negative effect of Fox. Panel B replicates panel A but adds controls for the channel positions of CNN and MSNBC. Neither of them appears to be significant and the coefficient for the Fox News Channel position lineup remains unchanged.^45^

We show the robustness of our results using alternative specifications in panel C and panel D. Panel C adds the interaction terms of the controls with the After dummy to take into account differential effects of socio-economic and political characteristics on social distancing. Panel D uses county fixed effects instead of state fixed effects to account for additional unobserved factors. The results are very similar to panel A.

We also check if our results are driven by some specific regions. (Ananyev et al. 2021) find that urban and rural areas did not respond differentially to the Fox exposure (columns VI–VII of Table A.1). In addition, it was not driven by some particular state (Figure A.3).

An alternative way to define the start of people’s awareness of the policy recommendation of social distancing is using states’ shelter-in-place orders rather than the national emergency. Suppose that states where voters had been more exposed to Fox also voted for the government that was later in issuing stay-at-home order. In addition, people follow these state-level shelter-in-place orders. Then our effect can be explained by people with more exposure to Fox decreasing their movement less because of the lagged timing of shelter-in-place policies. Ananyev et al. (2021) find similar results of Fox using the shelter-in-place order timings, suggesting that people are paying attention to both federal and state recommendations and that the state order timings are not endogenous with respect to Fox News Channel positions (Table A.3).

Ananyev et al. (2021) also consider heterogeneous effects in Table A.4. We find some evidence that exposure to Fox News had smaller effects in the locations with a higher share of the population with a high-school education and a higher share of the population employed in workable-from-home industries (columns IV and V). We find no differential effects in urban locations in column III or locations with a higher number of Christian churches in column VI (that we consider as a proxy to conservatism).^46^ We also find some suggestive evidence in columns VII and VIII, that counties that already reported first COVID cases and deaths also experienced smaller effect from the exposure to Fox News, suggesting that first-hand experience alleviated Fox mislead messages.

A natural extension would be to test the impact of Fox exposure on COVID cases and mortality rates. In Table A.5, Ananyev et al. (2021) document that locations more exposed to Fox experienced larger mortality rates from COVID-19, consistent with (Bursztyn et al. 2020). This suggests that Fox exposure can have important public health consequences through behavioral responses.

### Event study evidence

In the previous Section, we show results for non-dynamic specifications, where there is only one coefficient estimate for the Fox exposure for all dates after the National Emergency was announced.^47^ Alternatively, we allow separate point-estimates for weeks from February 24th to April 14th as follows:

2 $$ \begin{aligned} SD_{i(s)t(w)} = \underbrace{\sum\limits_{l=-4}^{-1} \gamma_{l} \cdot FNCP_{i(s)} \cdot \text{D}(w = l)}_{\text{pre-event period}} + \underbrace{\sum\limits_{l=0}^{4} \gamma_{l} \cdot FNCP_{i(s)} \cdot \text{D}(w=l)}_{\text{post-event period}} + \\ + X_{i(s)}{\Gamma} + \lambda_{t(w)} + \mu_{s} + \varepsilon_{i(s)t(w)}, \end{aligned} $$

where *SD*_*i*(*s*)*t*(*w*)_ is social-distancing outcome of county *i* in state *s* at date *t* in week *w*. Week *w* = 0 is the week of March 13 to March 20. Week indices run from − 4 to 4 and represent the position of weeks relative to week *w* = 0. D(*w* = *l*) is a dummy equal to one if week *w* = *l*. Here, *λ*_*t*(*w*)_ are date fixed effects and *μ*_*s*_ are state fixed effects. Coefficients *γ*_*l*_ with *l* ≥ 0 capture the Fox exposure effect in the post national emergency period, and the ones with *l* < 0 capture pre-trends.

**Fig. 4:**
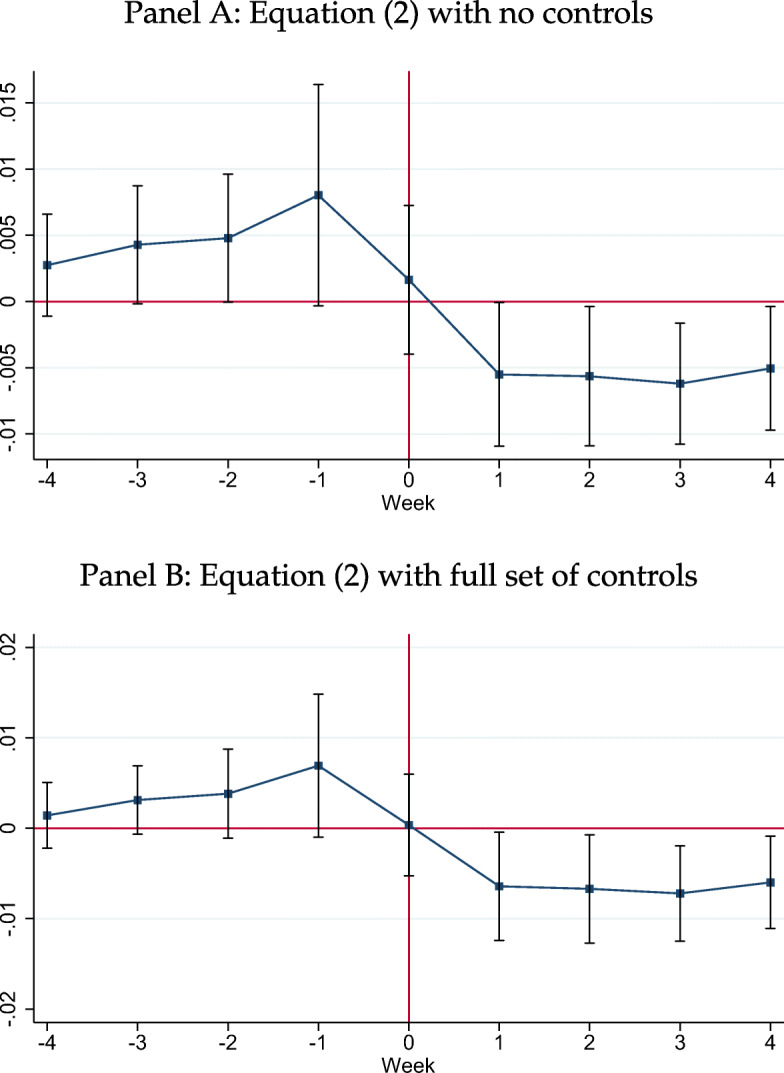
Event Study Analysis: No Changes in Distanced Traveled Before Week 0 and Large Reductions Afterwards. Note: This figure graphs the results of estimating Eq. 2 for the specifications in panel A of Table 2 without controls and with the full set of controls. The former corresponds to the specification in column 1 of Table 2. The latter corresponds to the specification in column 7 of Table 2. Point estimates are reported in Table A.6 of Ananyev et al. (2021). *p*-values for the joint significance of the pre-trend’s coefficients are equal to 0.403 for panel A and 0.448 for panel B. Following best practice, we bin the end-points, so that the fourth to the fifth week before and after March 13th each share a coefficient (Borusyak and Jaravel 2016; Schmidheiny and Siegloch 2019). This figure reports 90% confidence bands

Figure 4 plots the resulting coefficients of Eq. 2 for the specification without controls (panel A) and with the full set of controls (panel B).^48^ The first noteworthy feature is that neither specification exhibits pre-trends. There is an increase in the coefficient for the week prior to March 13th; however, the point estimate is insignificant. We fail to reject the joint *F*-test that the pre-event *γ*_*l*_s are zero. This suggests that the exact timing of the national emergency is not related to trends in social distancing in more-Fox-exposed counties and that social distancing behaviour did not start to change before the national emergency was announced.^49^

The second noteworthy feature is that while we do not observe any effect at the week zero (*γ*_0_), four point estimates for four weeks after March 13th have almost the same magnitude as the point estimate of $\widehat {\beta }_{2}$ from the baseline specification in Table 2. Thus, the effect is constant across all weeks and our baseline specification (1) captures the full time path of the effect. Ananyev et al. (2021) also show that the results also hold if one adds county fixed effects (Figure A.5, which is a similar specification to one in panel D of Table 2), using week *t* = − 1 as the baseline.

Ananyev et al. (2021) also replicate similar event-study graphs for the shelter-at-home orders in Figure A.4. Here, each state had its own relative time as week 0 started at the date when the state issued the order. While we see negative effects of the Fox News Channel position in the post-period, there are evident (while insignificant) downward pre-trends. This suggests that people might have started to decrease their mobility after the national emergency was announced but before their state officially ordered them to stay home.

### Zip-code-level results

Thanks to Facebook’s “Data for Good” project, we are able to investigate the effect of slant media on zip-code-level data for the subsample of 14 states and DC. Since the channel positions are initially on the zip-code level, we decrease potential measurement error.

We first confirm that county-level social distancing measures using Facebook data are highly correlated with measures using UNACAST data. Ananyev et al. (2021) report in Figure A.6 the residual plots of the regression of UNACAST’s changes in distance traveled on Facebook’s distance traveled (panel A) and Facebook’s probability of staying at home (panel B). In both graphs, the measures are strongly correlated. (Ananyev et al. 2021) also show that our baseline results in Table 2 hold if we use county-level Facebook measures (Table A.7).

Because Facebook’s data start on March 10th, we can’t estimate pre-trends as we did in the baseline specification. In addition, instead of the changes in mobility, we observe the levels of mobility in the Facebook data. Thus, we control for the pre-COVID mobility more flexibly using the following equation:

3 $$  M_{j(s)t} = \beta FNCP_{j(s)} +\phi M_{j(s)t-45} + X_{j(s)}{\Gamma} + \mu_{s} + \lambda_{t} + \epsilon_{j(s)t}, $$

where *M*_*j*(*s*)*t*_ is the mobility measure of zip code *j* in state *s* and date *t* and *M*_*j*(*s*)*t*− 45_ is the corresponding mobility measure in the 45 days before March 10. *FNCP*_*j*(*s*)_ is the Fox lineup position in zip-code *j* in state *s*. We again control for state and time fixed effects. Vector *X*_*j*(*s*)_ now contains zip-code-level controls, including the number of Facebook bing tiles covered, number of Facebook users, population, population density, number of housing units, and land area.

Here, we use two measures of mobility: (i) probability of staying at home (panel A of Table 3) and (ii) daily distance traveled (panel B). In panel A column 1, we only control for baseline probability of staying at home, number of tiles, Facebook’s population, and date fixed effects. Fox News Channel lineup position has positive effects on staying home: one standard deviation increase in channel position results in a 0.1-percentage-point larger probability of staying at home. Columns 2 and 3 add controls for Facebook’s measure of population density and state fixed effects. Column 4 allows for state-and-date fixed effects. Finally, columns 5–7 add controls for population, number of housing units, and land area. The coefficient of interest remains unchanged and highly significant throughout all columns.^50^ According to our results, among the 14 states (plus DC) where we have zip-code-level data, the 30-positions change in Fox increases the probability of staying at home by 0.2 percentage points. This explains 2% and 33% of the increase in the probability of staying at home in DC and West Virginia, respectively, which had the biggest and smallest changes.

**Table 3:** Zip-code-level evidence: more Fox News exposure, longer distance traveled, and smaller probability of staying at home

	(1)	(2)	(3)	(4)	(5)	(6)	(7)
Panel A:	Dependent variable: probability staying at home						
Fox News Channel	0.001***	0.001***	0.001***	0.001***	0.001***	0.001***	0.001***
position	(0.0003)	(0.0003)	(0.0003)	(0.0003)	(0.0003)	(0.0003)	(0.0003)
R-squared	0.909	0.914	0.915	0.924	0.924	0.924	0.925
Observations	85,511	83,004	83,004	83,004	83,004	83,004	83,004
Panel B:	Dependent variable: distance traveled						
Fox News Channel	–0.005	–0.005*	–0.008**	–0.007*	–0.007*	–0.008**	–0.008**
position	(0.0032)	(0.0031)	(0.0037)	(0.0037)	(0.0038)	(0.0038)	(0.0037)
R-squared	0.921	0.924	0.926	0.943	0.943	0.943	0.944
Observations	84,818	82,311	82,311	82,311	82,311	82,311	82,311
FEs: date	✓	✓	✓				
Population density		✓	✓	✓	✓	✓	✓
FEs: state			✓				
FEs: date x state				✓	✓	✓	✓
Population					✓	✓	✓
Housing units						✓	✓
Land area							✓

The explanatory variable in both panels is normalized to mean zero and standard deviation one. All regressions include date fixed effects, the number of tiles used to construct the dependent variable at date *t*, number of Facebook users in a county, and the baseline (pre-COVID) dependent variable constructed using corresponding tiles for date *t*. In parentheses, we report standard errors clustered on zip-code level. ***p< 0.01, **p< 0.05, *p< 0.1

Panel B reports results for the distance traveled. We, also find results consistent with our findings on the county-level: a one-standard-deviation increase in channel lineup explains 2.5% of differences in distance traveled between crisis and baseline measures. Overall, we find consistent evidence that Fox negatively affected social distancing responses both at the county and at the zip-code level.

## Conclusion

During an outbreak of a contagious disease, public behavior is extremely important, since every policy and each piece of advice from experts can only make a difference if they are followed, and followed by a substantial amount of people. The messages conveyed by media can either help or hinder these practices. In this paper, we estimate the effect of exposure to one popular media source (Fox)—that spread controversial partisan opinions and some unscientific medical advice during the early days of the COVID-19 pandemic—on mobility reduction and social distancing. Using county-level mobility data from smartphone locations and the historical position of Fox News Channel in the cable lineup, we show that increased exposure to Fox News led to a smaller reduction in distance traveled and a smaller increase in the probability to stay home after the national emergency declaration in the USA. We find that the results are not driven by the conservatism itself, measures as the Republican vote share, but come from the COVID-19-related information conveyed by Fox and its potential interaction with the built-up conservative ideology.

Exposure to Fox could have unintended political consequences as well. As Baccini et al. (2021) and Warshaw et al. (2020) point out, local COVID-19 cases harmed the popularity of the Republican party and Trump among the residents of affected areas. Thus, to the extent that Fox News influence could have contributed to excess mortality, the channel could have impacted the election results. The effects size that we document are moderate in magnitude, but given the political races in the USA are often decided by razor-thin margins in pivotal places, those effects could be just enough to tip the scales in favor of the Democrats in the 2020 Presidential elections.

Our findings are especially important in the era of increasing affective polarization (Rogowski and Sutherland (2016) and Boxell et al. (2020)). In this highly charged environment, any criticism of the current Republican administration from their Democratic opponents is often perceived as not being done in good faith regardless of its merits, triggering a defensive reaction from conservative media. None other than Fox host Tucker Carlson explained, on March 10, 2020, the logic of some conservative politicians and media personalities:

Maybe they’re just not paying attention, or maybe they believe they’re serving some higher cause by shading reality. ... Best not to say anything that might help the other side.^51^

This alleged desire *not to say anything that might help the other side* may impact politics, economic growth, and lives, which are all highly interconnected as we have witnessed in the current COVID-19 pandemic and expect to see in its aftermath.

## Footnotes

1 Scholars have shown that slanted media have an impact on voting and political preferences (DellaVigna and Kaplan 2007; Enikolopov et al. 2011; Adena et al. 2015), collective actions (Zernike 2010), political polarization (Prior 2007; Martin and Yurukoglu 2017), investment decisions (Friebel and Heinz 2014), judicial decisions (Ash and Poyker 2019), city budgets (Galletta and Ash 2019), and candidate entry (Arceneaux et al. 2020).

2 BusinessWire. “FOX Business Network to Launch on Channel 43 on Time Warner Cable. Fox News Channel Moves to Channel 44”, September 5, 2007. URL: www.businesswire.com/news/home/20070905006114/en/FOX-Business-Network-Launch-Channel-43-Time.

3 In Ananyev et al. (2021) Figure A.7 and A.8, we present empirical evidence documenting these endogenous changes in Fox News Channel positions from 2005 to 2015.

4 From our reading of Simonov et al. (2020) and Ash et al. (2020), they don’t control for the Republication vote shares. Ash et al. (2020) include political controls in their regression in the form of dummy variables for above-median Republican vote share in 2012 and 2016. Simonov et al. (2020) do a subsample analysis divided by the Republication vote shares, and we think it is hard to interpret the subsample findings. In any case, due to the fact that the 2005 to 2015 channel position changes were likely to be endogenous to the lobbying efforts, we think our approach can more cleanly isolate the information channel.

5 See, for example, https://colab.research.google.com/drive/1u15afRytJMsizySFqA2EPlXSh3KTmNTQ#sandboxMode=true&scrollTo=tnnbc9nMWxV1.

6 Ash et al. (2020) is less susceptible to this issue than Simonov et al. (2020) to the extent Ash et al. (2020) aggregate CBG-level data to a county-level.

7 According, to Pew Research Center, in 2020, 79% of people 18–49 years old, 68% of people 50–64, and 46% of those older than 65 used Facebook. When it comes to the urban-rural division, 73% of urban residents and 66% of rural residents used Facebook. See https://sproutsocial.com/insights/new-social-media-demographics/#.

8 See a detailed report in www.unacast.com/resources/how-accurate-is-unacast-data.

9 There are other notable differences in specification choices from Simonov et al. (2020): we use a reduced-form specification with date fixed effect, and they use two-stage least squares (2SLS) specification with time-invariant Fox viewership as the treatment variable.

10 WHO COVID-19 Timeline. URL: who.int/news-room/detail/27-04-2020-who-timeline---covid-19.

11 CDC. URL: cdc.gov/coronavirus/2019-ncov/cases-updates/cases-in-us.html (accessed on 28/04/2020).

12 CDC. URL: cdc.gov/coronavirus/2019-ncov/prevent-getting-sick/social-distancing.html(accessed on 28/04/2020).

13 See http://www.whitehouse.gov/presidential-actions/proclamation-declaring-national-emergency-concerning-novel-coronavirus-disease-covid-19-outbreak/http://www.whitehouse.gov/presidential-actions/proclamation-declaring-national-emergency-concerning-novel-coronavirus-disease-covid-19-outbreak/.

14 See www.cnbc.com/2020/03/13/trump-will-hold-a-press-conference-at-3-pm-et-to-discuss-coronavirus-response.html.

15 “The Dow Jones Industrial Average closed 1,985 points higher, or 9.4%, at 23,185.62., the index’s biggest-ever point gain on a single day.” See www.cnbc.com/2020/03/12/futures-stock-market-coronavirus-concerns.html.

16 The COVID-19 situation was evolving fast in the first few months of 2020, and given the nature of the novel disease, everyone experienced fast and extensive learning then. We think that during the March and April of 2020, what information was shared and how information was transmitted via different channels were especially important, and both the government’s action (in our case, the declaration of national emergency) and media influence (in our case, Fox News contents) were powerful in shaping people’s beliefs and affecting their actions. Later, many more people’s life was directly affected by the pandemic, and their own experience may become more important than outside information sources. In addition, many other important events happened in May and later months in the USA (for example, the aftermath following George Floyd’s death), which makes isolating the effect of each source more difficult. Overall, we view our study as capturing the effect of media on people’s pandemic responses in the short run.

17 Foxnews.com: “Fox News reaches highest viewership...”, URL: https://www.foxnews.com/media/highest-viewership-network-history-msnbc-cnn-2020.

18 Fox News Network Fox Hannity 9:00 pm EST March 12, 2020 Thursday. Source: Nexis Uni database.

19 Fox News Network Ingraham Angle 10:00 pm EST February 18, 2020 Tuesday. Source: Nexis Uni database.

20 Fox News Network Tucker Carlson Tonight 8:00 pm EST March 12, 2020 Thursday. Source: transcripts of Tucker Carlson Tonight from Nexi Uni database.

21 Not everyone at Fox was dismissive of the dangers of COVID-19. For example, Tucker Carlson warned his viewers several times during the early days of the disease and even seemed to criticize his Fox colleagues (though he also spent significant time critically discussing state-level lockdown policies). See, for example, Fox News Network Tucker Carlson Tonight 8:00 pm EST from February 27, 28, and March 11. Source: Nexis Uni database.

22 Mediaite.com, “Fox & Friends...” URL: http://www.mediaite.com/tv/fox-friends-churns-out-insane-misinformation-on-coronavirus/http://www.mediaite.com/tv/fox-friends-churns-out-insane-misinformation-on-coronavirus/.

23 Fox News Network Fox Hannity 9:00 pm EST March 9, 2020, Monday, Source: Nexis Uni database.

24 Fox News Network Ingraham Angle 10:00 pm EST, Source: Nexis Uni Database.

25 Washington Post: “How Fox News has shifted its coronavirus rhetoric,” www.youtube.com/watch?v=ifKbwDf51bA).

26 See msnbc.com/transcripts/rachel-maddow-show/2020-03-09.

27 See https://edition.cnn.com/2020/04/13/world/cnn-coronavirus-coverage/index.html.

28 We describe construction of the main variables in the next Section.

29 The first-stage relationship between channel lineup and Fox viewership on the zip-code level is in Table 2 of Martin and Yurukoglu (2017). County-level relationship can be seen in Supplementary Materials Figure 1 of Ash and Poyker (2019).

30 Ananyev et al. (2021) shows the distribution of the Fox channel positions on the county-level in Figure A.1.

31 Although we do not have the viewership data in 2020, Ananyev et al. (2021) provide evidence on the impact of 2005 channel positions on 2018 viewership using the DMA-level data in Figure A.9.

32 UNACAST reports daily distances travelled for those counties where there are at least 100 devices active on that date. There are 3054 counties in UNACAST data. Thus, daily measures have at least 100 smartphones per county per day and on average 4911 smartphones per day per county.

33 https://dataforgood.fb.com/docs/covid19/.

34 The 14 states include Arizona, California, Delaware, Idaho, Maryland, Montana, Nevada, New Jersey, Oregon, Pennsylvania, Utah, Virginia, Washington, and West Virginia. Some of these states are only partially in the data. E.g., we only have east of Pennsylvania, from Harrisburg to the border with New Jersey.

35 There are on average 2129 smartphones active per zip-code per day. The standard deviation is 3080, the minimum is 10 devices, and the maximum is 41,167 devices active per zip-code per day.

36 For details, see https://docs.microsoft.com/en-us/bingmaps/articles/bing-maps-tile-system.

37 Note that distance traveled is zero if a person stays at the same tile.

38 Results hold if we cluster by county or double-cluster by state and date or county and date. Clustering by state yield the most conservative standard errors.

39 Note that the dummy for Before and After sum up to one, and our specification is equivalent to including the Fox levels and the interaction of Fox with the After dummy. We present the results using our current specification to show the absence of pre-trends more straightforwardly.

40 Our design is not a difference-in-difference specification since all people in the U.S. received the National Emergency declaration on the same day, and had exposure to Fox News both before and after the declaration. Our focus is the coefficient *β*_2_, and *β*_1_ acts as a placebo test.

41 These political economy outcomes are measured after 2005. Thus, they can be affected by the Fox exposure set in 2005. We use these variables to highlight the mechanism, and we find that adding these controls does not affect the coefficient estimate of the Fox exposure, pointing towards the importance of COVID-related direct information transmission.

42 In principle, it could be the case that the conservatism ideology can also have an impact on economic outcomes, which affect social distancing behaviors. However, we do not find direct evidence supporting this hypothesis. As shown in Table 1, the 2005 Fox News Channel positions are not correlated with the 2010 demographic and socio-economic conditions.

43 In addition, we show that shares of workable-at-home jobs do not correlate with the Fox News Channel position (see Table 1). Ananyev et al. (2021) show robustness to alternative measures of workable-from-home employment in Table A.1.

44 Ananyev et al. (2021) include these three variables one-by-one (in columns II–IV of Table A.2). Inclusion of each of these controls separately does not affect the magnitude of the coefficient of interest.

45 It might seem puzzling why the positions of CNN and MSNBC yield non-significant coefficients given that their coverage differed from the Fox. It is important to point out, however, that only the initial location of the channels is plausibly exogenous. We are measuring the lineup in 2005, 25 years after the founding of CNN, thus it is not exogenous anymore. Thus, while controlling for CNN allows accounting for an outside option of viewing CNN, the coefficient on the endogenous control variable cannot be interpreted causally (Westreich and Greenland 2013). MSNBC’s channel position is arguably as plausibly exogenous as the Fox News one, and while the coefficient is of a correct sign, its non-significance is indeed surprising. One of the potential explanations is that the messages have asymmetric effects: messages designed to promote health-related precautions are less likely to be consequential than messages of skepticism and the ones the prime unrelated-to-health issues. As our focus in this paper is Fox News, we leave a more thorough exploration of the role of MSNBC for further research.

46 Heterogeneous results with respect to the share of votes for Republicans in 2016 and 2012 elections in columns I and II yield inconclusive results as both vote shares and social distancing are affected by the Fox News Channel position in 2005.

47 We prefer the National Emergency declaration specification for the following reasons. While everyone received the same message at the same time through the national emergency declaration, different states issued stay-at-home on different dates. It could be the case that the national signal was already enough to initiate people’s responses by reducing their mobility, and state-level stay-at-home orders reinforced it. It could also be the case that states with higher Fox exposure chose to issue the stay-at-home order later since the state government also viewed the COVID severity more lightly, or chose to issue the order earlier since the local outbreak was more severe. Due to these two concerns, we decide that the national emergency declaration was a cleaner point in time to expect people’s responses.

48 The coefficients in Fig. 4 can be viewed as period-by-period regression coefficients, and no week is specified as the baseline week. This is consistent with our main specification in Table 2 panel A.

49 These specifications correspond to column 1 and column 7 of Table 2 panel A. Point estimates for Fig. 4 are reported in Table A.6 of Ananyev et al. (2021).

50 Ananyev et al. (2021) Table A.8 presents results using alternative clustering by a service provider, and the results are similar.

51 Foxnews.com: “Tucker Carlson: The Coronavirus Will Get Worse...,” URL: foxnews.com/opinion/tucker-carlson-the-coronavirus-will-get-worse-our-leaders-need-to-stop-lying-about-that.
